# Primary Breast Lymphoma: A Rare Clinical Entity

**DOI:** 10.4021/wjon319w

**Published:** 2011-06-08

**Authors:** Regina S. Offodile, Veronica Arce, Jessica Cross, Jarvis Reed, Derrick J. Beech

**Affiliations:** a Department of Surgery, Meharry Medical College, School of Medicine, USA; b Department of Surgery/Section of Surgical Oncology, University of Tennessee Health Science Center, College of Medicine, USA

**Keywords:** Lymphoma, Breast cancer, Mammary

## Abstract

Localized primary breast lymphoma is very rare. The typical clinical and radiographic presentation of isolated primary breast lymphoma mimics that of breast adenocarcinoma. Histologic diagnosis of primary breast lymphoma relies heavily on Hematoxylin and Eosin pathologic evaluation and immunohistochemical staining. Cytotoxic systemic chemotherapy is the primary treatment for this disease with the occasional need for adjuvant radiation therapy or surgical resection. This case report outlines the diagnosis and management of a patient with primary breast lymphoma.

## Introduction

Primary breast lymphoma is a rare clinical entity representing less than 1% of all breast malignancies [1]. Primary breast lymphoma (PBL) was defined by Wiseman and Liao as any lesion that had an adequate pathologic specimen; both mammary tissue and lymphomatous infiltrates in closer relation to each other; no evidence of widespread disease and no prior diagnosis of extra mammary lymphoma. Approximately 54,000 patients are diagnosed annually with non-Hodgkin’s lymphoma with disease isolated to the breast occurring in less than 1% of all patients [2]. Among patients with extranodal manifestations of non-Hodgkin’s lymphoma, primary breast lymphoma occurs in approximately 2% of this cohort [2].

Majority of breast malignancies are adenocarcinoma with the rare occurrence of other histologic subtypes such as soft tissue sarcoma [3]. As with breast adenocarcinoma, the diagnosis of breast lymphoma is dependent on radiographic and ultimately pathologic criteria. The diagnostic workup frequently includes mammography and breast ultrasounds and biopsy of the mass. This case report highlights the relevant diagnostic and therapeutic components involved in the management of patients with primary breast lymphoma.

## Case Report

A 68-year-old woman was referred to the surgical oncology service after presenting the initial complaint of a palpable mass in her left breast discovered on breast self examination. She reported initially discovering the mass approximately two months prior to presentation. She denied the presence of previous breast nodules, skin changes, nipple drainage or nipple retraction. The mass was reported to be approximately the size of a grape when initially discovered and has increased significantly in size over the last several weeks. Patient denied the history of breast trauma.

The patient is postmenopausal, gravida 2, para 2 and taking exogenous hormones to control menopausal symptoms. She did not report a history of previous breast biopsy. Patient denied a family history of breast cancer. Reviews of systems were otherwise unremarkable.

Physical examination demonstrated symmetric breasts without evidence of breast skin changes or nipple retraction. There were no palpable masses of her right breast and a 4 centimeter dominant mass at the 12 o’clock position of her left breast. The mass was mobile and non-tender to palpation. There was no evidence of axillary or supraclavicular lymphadenopathy. Examination of other lymph node basins demonstrated no evidence of lymphanopathy.

Diagnostic studies included bilateral mammography and left breast ultrasound Mammography did not demonstrate any suspicious changes or findings of the right breast. There was a smooth-bordered density centrally located in the upper portion of the patient’s left breast that was highly suspicious for malignancy. Ultrasound evaluation confirmed a solid mass.

**Figure 1:**
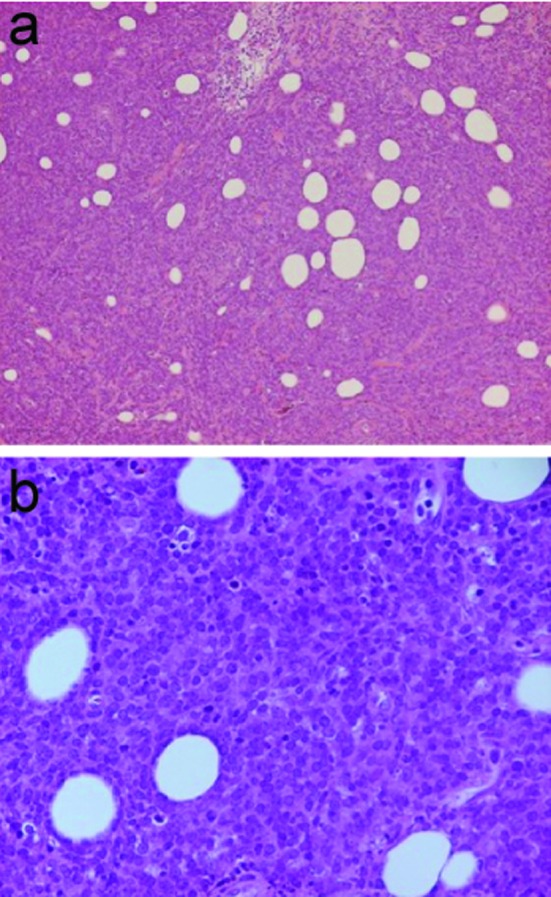
Hematoxylin and Eosin stain, (a) 10 X and (b) 40 X magnification, demonstrating atypical mononuclear large cell infiltrate with poorly defined cytoplasmic borders, large prominent nucleoli and frequent mitosis.

**Figure 2:**
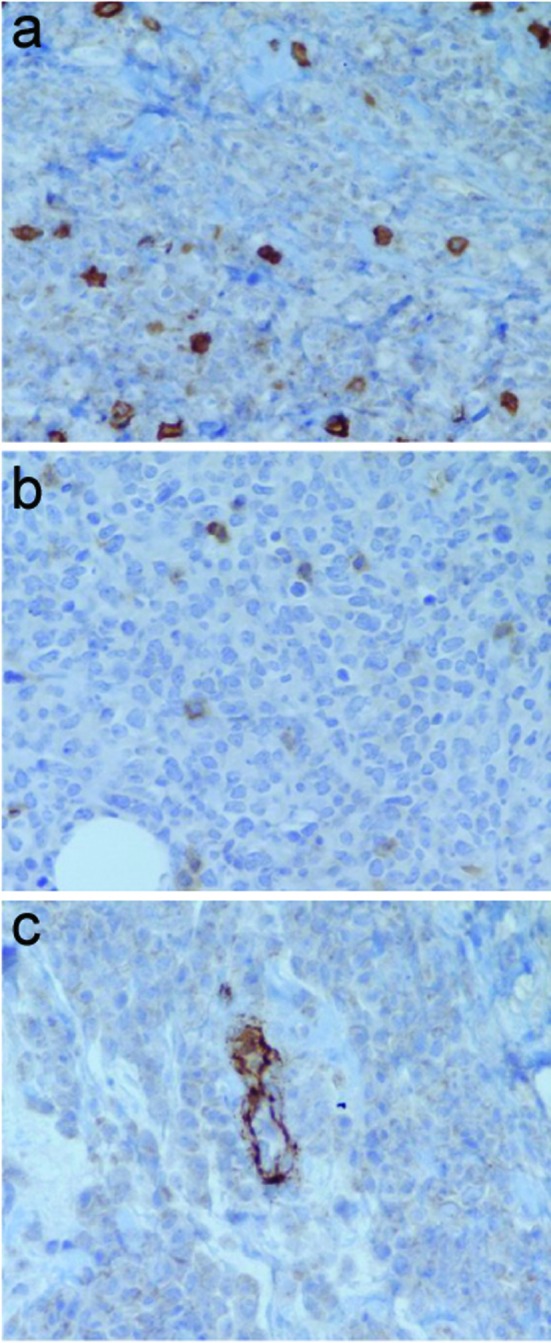
Immunoperoxidase staining negative for (a) CD 3, (b) CD 5, and (c) CD 34.

**Figure 3:**
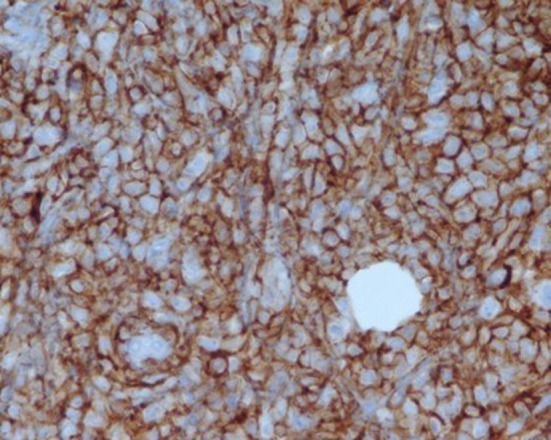
Positive immunoperoxidase staining for CD 20 (60 X magnification).

The patient underwent an ultrasound-guided needle biopsy which demonstrated left breast lymphoma. An incisional biopsy was performed to confirm the detailed histologic classification. The surgical pathology report confirmed findings of an atypical mononuclear large cell infiltrate with poorly defined cytoplasmic borders, large, prominent nucleoli, and frequent mitoses (Fig. 1). Immunohistochemical staining was positive for CD20 immunoperoxidase stain and, in a small portion of the tumor cells, Ki-67 immunoperoxidase stain. CD3, CD5, CD10, CD34, and TdT immunoperoxidase stains were all negative (Fig. 2). Based on morphology and immunoperoxidase staining, the final diagnosis of diffuse large B-cell lymphoma was made (Fig. 3). The clinical and pathologic findings were discussed with a multidisciplinary team. The decision was made to proceed with cytotoxic chemotherapy with consideration of salvage surgical management following the completion of treatment if there was residual disease.

## Discussion

Isolated breast lymphoma occurs in less than 1% of all patients with non-Hodgkin’s lymphoma. Clinical presentation for patients with breast lymphoma is very similar to that of patients that present with other mammary neoplasia. The typical clinical presentation is that of a palpable breast mass found on self-examination or clinical breast examination. In fact, unlike the current patterns by which adenocarcinoma of the breast are detected, breast lymphomas are most likely to be discovered initially due to a palpable mass on breast self-examination or clinical breast examination. Domchek and associates reported that more than 90% of patients with breast lymphoma presented with a palpable mass [2]. The increased use of screening mammography has not led to a proportional increase in the diagnosis of primary breast lymphoma nor has it translated into earlier detection of patients with breast lymphoma [2]. As with our patients, it is commonly noted that the lesion progressed in size rapidly after initial detection. The rapid increase in size is not typical of breast adenocarcinoma as suggested by the possibility of lymphoma.

The initial description of clinical criteria for defining primary breast lymphoma was reported by Wiseman and Liao in 1972 [4]. These authors suggested that the criteria for clinical classification of primary breast lymphoma depended on the histologic confirmation of disease along with associated mammary tissue in close alignment with a lymphomatous infiltration and no clinical evidence of diffuse involvement of other lymph node basin or extra nodal sites. Also, patients with primary breast lymphoma must not have a previous history of lymphoma.

The diagnostic evaluation for lymphoma is similar to that for breast adenocarcinoma. The mammography and breast ultrasound are useful techniques in evaluating the remainder of the ipsilateral breast as well as the contralateral breast. Once an abnormal finding is confirmed by clinical exam and radiographic studies histologic confirmation of lymphoma is required.

Domchek and associates reported that nearly 91% of all patients with diagnosed primary breast lymphoma discovered the disease during clinical breast examination or breast self examinations [2]. Once histologic confirmation of lymphoma was performed either by core needle biopsy techniques or open biopsies, patients were then categorized based on their cell type of lymphoma. Diffuse large cell lymphoma was the most common histologic subtype discovered in patients with primary breast lymphoma. These tumors are typically characterized by the presence of large cells with typically vesicular nuclei, scant cytoplasm and variably prominent or discernable nucleoli. Patients may also demonstrate histologic findings compatible with low grade follicular lymphoma. Typical B-cell phenotypes showed reactive findings to CD10 cell origin. The status of CD5, CD10, CD43, and the morphologic characteristics of these cells allow the specific categories of lymphoma to be determined. That Burkitt-like lymphomas may also be cleaved cell type lymphoma was also noted to be present in a small number of patients. These tumors demonstrated CD20 reactivity on immunohistochemistry and were of B-cell present in patients with primary breast disease. These tumors typically display a T (8:14) chromosomal translocation which is characteristics of Burkitt’s lymphoma. They may also demonstrate expression levels that are detectable for the Epstein-Barr virus associated antigen.

As previously noted, there are a variety of histologic subtypes of lymphoma that can exist in primary breast lymphomatous disease. The most common subtype is that of diffuse large cell non-Hodgkin’s lymphoma. Primary breast lymphoma can also exhibit features typical of MALT (mucosal associated lymphoid tissue) lymphoma. Burkitt-like lymphoma is typically thought to be associated with pregnancy [5].

Treatment of primary breast lymphoma typically involves excision of the lesion if successful excising can be performed without deforming the breast contour.

Wiseman and Liao reported dismal results of patients with primary breast lymphoma treated with surgery alone or those treated with radiation therapy [4]. Specifically, the survival was only 3 out of 16 patients. Survival rates have been markedly improved by the addition of systemic cytotoxic chemotherapy. Mastectomies may occasionally be performed; however, these lesions are responsive to cytotoxic systemic chemotherapy. As such, mastectomy can typically be avoided in the majority of patients [6]. Patients with high grade lymphomas or immediate grade lymphomas are typically treated with cytotoxic chemotherapy with or without the addition of external beam radiation therapy. Many of these patients will not ultimately require surgical ablation. Radiation therapy alone is typically inadequate in controlling the disease for primary breast lymphoma [6, 7]. Radiation therapy, however, can be occasionally beneficial when used as adjuvant therapy following chemotherapy.

Primary breast lymphoma is a rare clinical entity with disease typically noted with a palpable breast mass discovered by the patient or during clinical breast examination by the health care provider. The use of traditional radiographic methods, including mammography and ultrasound, may confirm the presence of an abnormal lesion. Histologic confirmation demonstrates several varieties and cell types of non-Hodgkin’s lymphoma. Diffuse large cell lymphoma is the most commonly diagnosed form of primary breast lymphoma. Several other histologic subtypes may occur. Immunochemistry and immunoperoxidase staining is essential in demonstrating a subtype lymphoma as is flow cytometry. Cytotoxic chemotherapy remains the primary means by which these lesions are treated. Radiation therapy can also be of benefit when used in an adjuvant setting. Many patients can avoid mastectomy if systemic chemotherapy is used as the initial form of treatment. Survival in patients treated with cytotoxic chemotherapy has improved over the last several years.
